# High-throughput sequencing revealed differences of microbial community structure and diversity between healthy and diseased *Caulerpa lentillifera*

**DOI:** 10.1186/s12866-019-1605-5

**Published:** 2019-10-15

**Authors:** Zhourui Liang, Fuli Liu, Wenjun Wang, Pengyan Zhang, Xiutao Sun, Feijiu Wang, Heather Kell

**Affiliations:** 1 Key Laboratory of Sustainable Development of Marine Fisheries, Ministry of Agriculture and Rural Affairs, Yellow Sea Fisheries Research Institute, Chinese Academy of Fishery Sciences, Qingdao, China; 2 Laboratory for Marine Fisheries Science and Food Production Processes, Qingdao National Laboratory for Marine Science and Technology, Qingdao, China; 3 College of Science and Engineering, Flinders University, Adelaide, Australia

**Keywords:** *Caulerpa lentillifera*, High-throughput sequencing, Microbial community, Diversity, Diseased

## Abstract

**Background:**

*Caulerpa lentillifera* is one of the most important economic green macroalgae in the world. Increasing demand for consumption has led to the commercial cultivation of *C. lentillifera* in Japan and Vietnam in recent decades. Concomitant with the increase of *C. lentillifera* cultivation is a rise in disease. We hypothesise that epiphytes or other microorganisms outbreak at the *C. lentillifera* farm may be an important factor contributing to disease in *C. lentillifera*. The main aims are obtaining differences in the microbial community structure and diversity between healthy and diseased *C. lentillifera* and key epiphytes and other microorganisms affecting the differences through the results of high-throughput sequencing and bioinformatics analysis in the present study**.**

**Results:**

A total of 14,050, 2479, and 941 operational taxonomic units (OTUs) were obtained from all samples using 16S rDNA, 18S rDNA, and internal transcribed spacer (ITS) high-throughput sequencing, respectively. 16S rDNA sequencing and 18S rDNA sequencing showed that microbial community diversity was higher in diseased *C. lentillifera* than in healthy *C. lentillifera*. Both PCoA results and UPGMA results indicated that the healthy and diseased algae samples have characteristically different microbial communities. The predominant prokaryotic phyla were Proteobacteria, Planctomycetes, Bacteroidetes, Cyanobacteria, Acidobacteria, Acidobacteria and Parcubacteria in all sequences. Chlorophyta was the most abundant eukaryotic phylum followed by Bacillariophyta based on 18S rDNA sequencing. Ascomycota was the dominant fungal phylum detected in healthy *C. lentillifera* based on ITS sequencing, whereas fungi was rare in diseased *C. lentillifera*, suggesting that Ascomycota was probably fungal endosymbiont in healthy *C. lentillifera*. There was a significantly higher abundance of Bacteroidetes, Cyanobacteria, Bacillariophyta, Ulvales and *Tetraselmis* in diseased *C. lentillifera* than in healthy *C. lentillifera*. Disease outbreaks significantly change carbohydrate metabolism, environmental information processing and genetic information processing of prokaryotic communities in *C. lentillifera* through predicted functional analyses using the Tax4Fun tool.

**Conclusions:**

Bacteroidetes, Cyanobacteria, Bacillariophyta, Ulvales and *Tetraselmis* outbreak at the *C. lentillifera* farm sites was an important factor contributing to disease in *C. lentillifera*.

## Background

*Caulerpa lentillifera*, also known as sea grape or green caviar, is a coenocytic green alga having a wide distribution in the tropical Indo-Pacific region [18, 50, 58, 67]. *C. lentillifera* is characterized by a thallus consisting of long horizontal stolons with many erect grapelike branches above and filiform rhizoidal branches below. The erect branches are populated with many small spherical ramuli, each tightly attached to the main axis [49]. *C. lentillifera* is a popular seafood delicacy in Japan, Korea, Philippines and other southeast Asian countries, eaten fresh or as a salt-preserved form. Its bright green color, delicate flavor, and soft and succulent texture make it highly sought after by consumers. Due to its nutritional and health value, with antibacterial and anti-inflammatory properties [53], it has received more attention in recent years and is rapidly becoming one of the most important economic green macroalgae in the world.

Increasing consumer demand has led to the commercial cultivation of *C. lentillifera* in Japan and Vietnam in recent decades. Cultivation methods vary and are adapted in different ways, depending on the country and site conditions. For instance, *C. lentillifera* is cultivated using a bottom-planting method in the Philippines [36], an off-bottom tray method in Vietnam, and a land-based raceway method in Japan [66]. The increasing demand for domestic consumption as well as international trade has promoted the commercial cultivation of *C. lentillifera* in China in recent years. Concomitant with the increase of *C. lentillifera* cultivation is a rise in disease associated with this species, particularly, invasion of epiphytes or bacteria.

In 2017, a disease outbreak occurred at a *C. lentillifera* farm in Dalian city with some obvious biofouling attachment on the surface of *C. lentillifera*. The spherical ramulis of the infected algae turned pink-red and detached from the diseased erect branches once the disease became severe, after which the infected algae decayed gradually*.* However, the causative agents and associated factors giving rise to the disease outbreak remain unclear.

Interactions among macroalgae and other attaching organisms including epiphyte and endophyte, such as bacteria and fungus, are complex. They can interact with each other, either synergistically or antagonistically. On the one hand, macroalgae harbor a rich diversity of associated microorganisms with functions related to host health and defense, which interact as a unified functional entity or holobiont [25]. Bacterial species and strains having similar metabolic functions were found to colonize similar algal taxa or algal groups [30]. Those bacteria with antifouling properties are thought to protect chemically undefended macroalgae from detrimental, secondary colonization by other microscopic and macroscopic epibiota [25]. On the other hand, the epiphytes and microorganisms may have negative effects to the macroalgae, including competition for nutrients, increasing the attachment and growth of a variety of other biofouling organisms, such as diatoms and other epiphyte algae spores, inhibiting gas exchange as well as reducing the availability of light and subsequent photosynthetic activity [20, 59].

Microorganisms are increasingly being recognized as the causative agents in the diseases of macroalgae [77] and epiphyte outbreaks have shown to weaken the seaweed, making it susceptible to bacterial attack [70]. Recent molecular studies have explored the epiphytic and bacterial diversity on some macroalgal species including *Caulerpa* [1, 6, 39, 68]. However, little is known of the microbial community structure and diversity for *C. lentillifera*.

A better insight into mutualistic interactions between macroalgae and other eukaryotes or prokaryotes is necessary for understanding and predicting algal disease outbreaks [30]. For studying prokaryotes, PCR amplification of the ubiquitous 16S ribosomal RNA (rRNA) gene is commonly used. Sequencing the variable regions of this gene allows precise taxonomic identification. For studying eukaryotic microbes such as fungi, as the equivalent rRNA gene (18S) may not provide sufficient taxonomic discrimination, the hypervariable internal transcribed spacer (ITS) is often used [69]. However, the 18S rRNA gene is more conserved and provides an independent measure of eukaryotic diversity that can identify biases in ITS analysis [54]. Hence, to determine the identity of the causal organism on the *C. lentillifera*, the prokaryotic and eukaryotic microorganism community structures and diversities of healthy and diseased *C. lentillifera* were explored using 16S rDNA, 18S rDNA, and ITS high-throughput sequencing in the present study. The use of high-throughput sequencing technologies has been widely adopted as they allow the identification of thousands to millions of sequences in a sample, revealing the abundances of even rare microbial species [69]. To the best of our knowledge, this is the first high-throughput amplicon sequencing study on the microbial community structure and diversity in *C. lentillifera*. The findings from such investigations may shed light on the cause and process of disease outbreaks in *C. lentillifera* and such knowledge would benefit the ability to control for disease under cultivation conditions.

## Results

### Richness and diversity

**Fig. 1:**
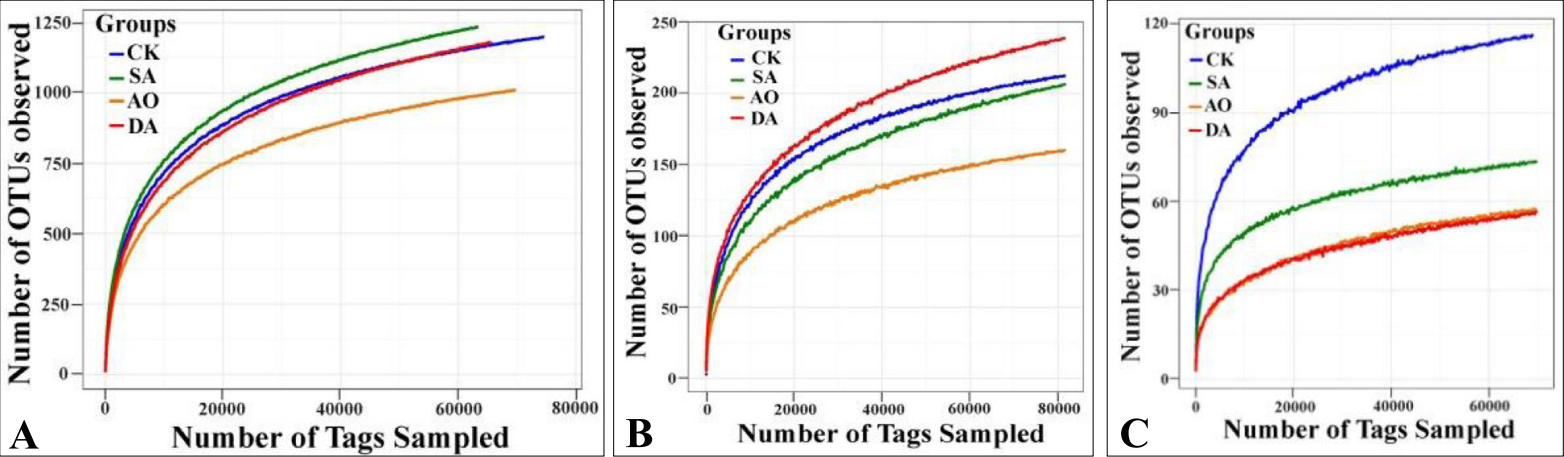
The rarefaction analysis of all samples. **a** 16S rDNA; (**b**) 18S rDNA; (**c**) ITS. Rarefaction curves of OTUs clustered for a dissimilarity of 3%. CK, SA, DA represent the healthy, diseased, and decayed algae samples respectively. AO represents the sediment samples collected from the algae farm

After filtering chimeric sequences and mismatches, the total number of V3-V4 region of the 16S rRNA gene reads, V4 region of the 18S rRNA gene reads, and ITS2 region reads obtained from the 12 samples, was 1,023,109, 1,559,260 and 1,171,931, respectively. They were respectively clustered into 14,050, 2479 and 941 OTUs at a cut-off of 97% sequence similarity, respectively. Rarefaction curves of most samples tend to be flat (Fig. 1), suggesting that a reasonable sequencing depth has been attained, although extra rare bacterial taxa are likely present in the sample. This was further supported by high Good’s coverage estimates (Table 1).

**Table 1:** Richness and diversity estimation of the 16S rDNA, 18S rDNA, and ITS sequencing libraries

Sample	OTUs	ACE	Chao1	Shannon	Simpson	Coverage(%)	Effective Tags
16S rDNA							
CK	1217 ± 254 a	1394 ± 219 a	1405 ± 212 a	6.921 ± 0.072 b	0.971 ± 0.004 a	99.730 ± 0.027 a	84,020 ± 5587 a
SA	1239 ± 56 a	1437 ± 89 a	1465 ± 92 a	7.570 ± 0.402 a	0.982 ± 0.010 a	99.614 ± 0.048 b	87,206 ± 2441 a
DA	1206 ± 198 a	1422 ± 198 a	1438 ± 196 a	6.909 ± 0.406 b	0.971 ± 0.011 a	99.645 ± 0.019 b	82,879 ± 8562 a
AO	1022 ± 72 a	1178 ± 79 a	1185 ± 76 a	6.614 ± 0.117 b	0.959 ± 0.002 b	99.734 ± 0.004 a	86,932 ± 5643 a
18S rDNA							
CK	214 ± 25 a	252 ± 43 b	244 ± 30 a	1.288 ± 0.258 c	0.264 ± 0.062 c	99.953 ± 0.009 ab	11,664 ± 3851 c
SA	209 ± 26 a	262 ± 13 ab	262 ± 20 a	2.729 ± 0.107 a	0.706 ± 0.073 a	99.940 ± 0.002 bc	11,934 ± 1426 c
DA	240 ± 7 a	291 ± 7 a	292 ± 4 a	3.182 ± 0.497 a	0.763 ± 0.053 a	99.934 ± 0.005 c	14,693 ± 8656 a
AO	163 ± 5 b	195 ± 22 c	195 ± 14 b	1.922 ± 0.066 b	0.478 ± 0.017 b	99.961 ± 0.009 a	13,682 ± 2948 b
ITS							
CK	118 ± 10 a	129 ± 1 a	133 ± 11 a	3.006 ± 0.110 a	0.785 ± 0.026 a	99.977 ± 0.002 a	77,855 ± 1035 c
SA	76 ± 5 b	88 ± 9 b	89 ± 8 b	2.423 ± 0.148 b	0.713 ± 0.028 a	99.984 ± 0.003 a	10,744 ± 2269 ab
DA	59 ± 6 c	78 ± 25 b	78 ± 1 b	1.219 ± 0.615 c	0.354 ± 0.211b	99.983 ± 0.006 a	94,962 ± 9216 b
AO	60 ± 5 c	69 ± 8 b	70 ± 9 b	1.692 ± 0.181 c	0.495 ± 0.040 b	99.986 ± 0.005 a	11,038 ± 6244 a

The cutoff value was 0.03 (sequence identity 0.97). ACE and Chao1 indices were used to evaluate the community richness, while Shannon and Simpson indices were used to assess the community diversity. The values of mean ± SD of three samples are shown in the table. The different letters superscript indicate significant differences. CK, SA, DA represent the healthy, diseased, and decayed algae samples respectively. AO represents the sediment samples collected from the algae farm

The average OTU numbers, community richness and community diversity of each group are shown in Table 1. Both the richness indices (including ACE index and Chao1 index) and diversity indices (including Shannon index and Simpson index) were higher in 16S rDNA groups than in 18S rDNA groups or ITS groups. The 16S rDNA and 18S rDNA OTUs detected in all algae groups (CK, SA, DA) were both more abundant than in the sediment group (AO). The OTUs and richness indices detected in CK were more abundant in the ITS groups. Moreover, all the community richness and diversity indices in all algae groups were higher than in the sediment group, indicating that additional OTUs are likely present in AO, although coverage estimates were very high for all samples. There was no significant difference in the richness indices (including Ace and Chao) between SA and DA in the same amplicon sequencing group (*p* > 0.05).

The Shannon index of SA in 16S rDNA groups and both the Shannon and Simpson indices of SA in 18S rDNA groups were significantly greater than those of CK (*p* < 0.05). There was a significantly greater Shannon index in SA compared to that of DA in 16S rDNA groups (*p* < 0.05). Moreover, both the Shannon and Simpson indices were significantly higher in SA than those of DA in ITS groups (*p* < 0.05). However, the richness index and Shannon index were both found to be significantly higher in CK than those of other groups based on ITS sequencing (p < 0.05).

### Prokaryotic community composition

**Fig. 2:**
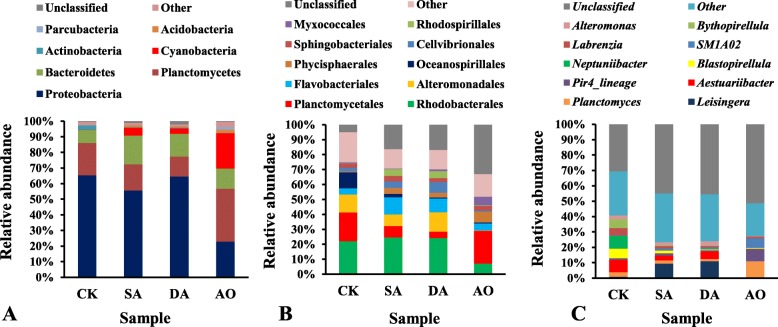
Relative abundance of predominant prokaryotes in all samples at three classification levels. **a** at the phylum level; (**b**) at the order level; (**c**) at the genera level. Sample abbreviations are as in Fig. 1. Planctomycetales, *Bythopirellula* and *Blastopirellula* belong to Planctomycetes, Planctomycetacia; Oceanospirillales, Cellvibrionales, *Aestuariibacter*, and *Neptuniibacter* belong to Proteobacteria, Gammaproteobacteria; Rhodospirillales, *Labrenzia*,and *Leisingera* belong to Proteobacteria, Alphaproteobacteria; Phycisphaerales and *SM1A02* belong to Planctomycetes, Phycisphaerae; Flavobacteriales belongs to Bacteroidetes, Flavobacteriia

The composition of prokaryotes at the phylum level was analyzed (Fig. 2a). Twenty-five prokaryotic phyla were detected in all samples, however, only seven of these phyla accounted for more than 96.9% of all sequences. The predominant phyla were Proteobacteria (52.1%), Planctomycetes (21.1%), Bacteroidetes (13.5%), Cyanobacteria (7.8%), Acidobacteria (1.0%), Acidobacteria (1.0%) and Parcubacteria (0.5%) in all sequences. The unclassified prokaryote at phylum level accounted for 1.0% of all sequences. Proteobacteria was the most predominant phylum, accounting for 65.3, 55.7, 64.6, and 22.9% of the reads in CK, SA, DA, and AO libraries respectively. Planctomycetes was the second most predominant phylum with proportions of 20.8, 16.7, 12.8, and 33.9% in CK, SA, DA, and AO respectively. The abundance of Bacteroidetes in CK was significantly lower than in SA and DA (*p* < 0.05). The abundance of Cyanobacteria in CK was very low, accounting for only 0.1% in CK and was significantly lower than in SA or DA (p < 0.05). Moreover, the abundance of Cyanobacteria in AO was very high, accounting for 22.7%.

The relative abundance of predominant prokaryotes at the order and genera level are shown in Fig. 2b and c respectively. The unclassified prokaryote at order and genera level accounted for 17.8 and 43.1% of all sequences respectively. Rhodobacterales and *Leisingera* were the most predominant order and genera respectively. Rhodobacterales accounted for 22.0, 24.5, 24.3, and 7.1% of the reads, and *Leisingera* accounted for 0.8, 9.5, 11.0, and 0.4% of the reads in CK, SA, DA, and AO libraries respectively. The abundance of Planctomycetales, Oceanospirillales, *Aestuariibacter*, *Neptuniibacter*, *Labrenzia*, *Bythopirellula*, and *Blastopirellula* in CK were all significantly higher compared to those in SA or DA (*p* < 0.05). Conversely, the abundance of Flavobacteriales, Phycisphaerales, Cellvibrionales, Rhodospirillales, *Leisingera*, and *SM1A02* in CK were all significantly lower compared to those in SA or DA (p < 0.05).

### Eukaryotic community composition based on 18S rDNA sequencing

**Fig. 3:**
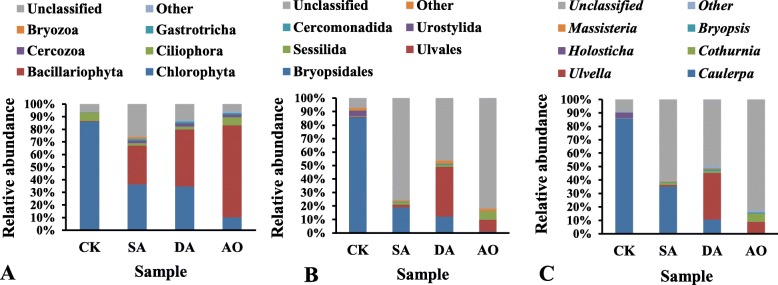
Relative abundance of predominant eukaryotes in all samples at three classification levels based on 18S rDNA sequencing. **a** at the phylum level; (**b**) at the order level; (**c**) at the genera level. Sample abbreviations are as in Fig. 1. Ulvales and *Ulvella* belong to Chlorophyta

Figure 3 shows the composition of eukaryotes at the phylum, order and genera level based on 18S rDNA sequencing. Sixteen eukaryotic phyla were detected in all samples based on 18S rDNA sequencing, however, only six of these accounted for about 86.7% of all sequences. The predominant phyla were Chlorophyta, Bacillariophyta, Ciliophora, Cercozoa, Gastrotricha and Bryozoa, among which Plantage and Animalia accounted for about 79.2 and 7.5% of all sequences respectively. Within the fungal domain, Ascomycota and Basidiomycota were detected, but the abundance of both was negligible in the samples. The unclassified eukaryote at phylum and genera level accounted for 12.4 and 50.1% of all sequences respectively based on 18S rDNA sequencing. Chlorophyta was the most predominant phylum, making up 86.0, 36.2, 34.6, and 10.1% of the reads in CK, SA, DA, and AO libraries respectively. Bacillariophyta was the second most predominant phylum, which accounted for 0.6, 30.8, 45.4, and 73.1% in CK, SA, DA, and AO respectively. The abundance of Bacillariophyta in CK was significantly lower than in other groups (*p* < 0.05), while the abundance of Ciliophora, Urostylida and *Holosticha* (belonging to Protozoa) in CK were all significantly higher than in SA and DA (p < 0.05). Ulvales were the dominant order, which accounted for 37.0% in DA, while only making up 0.3 and 2.0% of the reads in CK and SA respectively. Moreover, at the genera level, *Ulvella* accounted for 34.7% in DA but only accounted for 0.1 and 1.1% in CK and SA respectively. Thus implying that Ulvales or *Ulvella* grew abundantly on *C. lentillifera* when *C. lentillifera* decayed.

### Eukaryotic community composition based on ITS sequencing

**Fig. 4:**
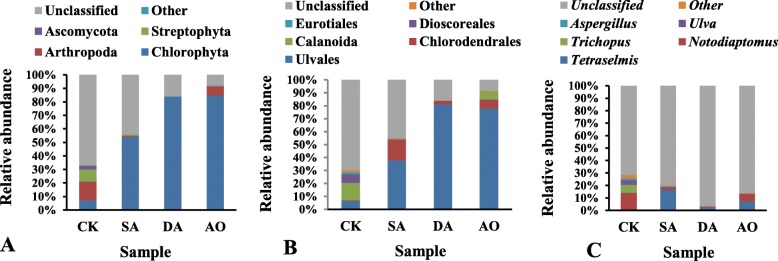
Relative abundance of predominant eukaryotes in all samples at three classification levels based on ITS sequencing. **a** at the phylum level; (**b**) at the order level; (**c**) at the genera level. Sample abbreviations are as in Fig. 1. Ulvales and *Tetraselmis* belong to Chlorophyta. Aspergillus belongs to Ascomycota

The relative abundance of predominant eukaryotes at the phylum, order and genera level based on ITS sequencing were shown at Fig. 4. Only five eukaryotic phyla were detected in all samples and the unclassified eukaryote at phylum, order and genera level accounted for 33.9, 34.8 and 83.6% of all sequences respectively. Unlike the result of 18S rDNA sequencing for the fungal domain, Ascomycota was one of the dominant phyla based on ITS sequencing, the abundance (2.7%) of which was significantly higher in CK than in the other groups (*p* < 0.05). Moreover, *Aspergillus*, a genus of fungi in the order Eurotiales (phylum Ascomycota), was also significantly higher in CK than in the other groups (*p* < 0.05), but *Aspergillus* could not be detected in AO based on ITS sequencing. In similarity with the result of 18S rDNA sequencing at the order level, Ulvales were the dominant order. The abundance of Ulvales in CK was significantly lower than in the other groups (*p* < 0.05), while the abundance of Arthropoda, Calanoida and *Notodiaptomus* (belonging to Metazoa) in CK were all significantly higher than in SA and DA (p < 0.05). At the genera level, *Tetraselmis*, a genus of green microalga, accounted for 16.1% in SA but only accounted for 0.2 and 2.6% in CK and DA respectively.

### Relationships among the microbial communities in the different samples

**Fig. 5:**
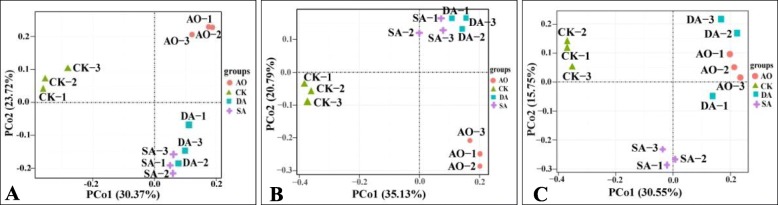
Principal Coordinates Analysis (PCoA) results showing the relatedness of microbial communities in the different samples. The PCoA plots were constructed with the unweighted UniFrac PCoA method. **a** 16S rDNA; (**b**) 18S rDNA; (**c**) ITS. Sample abbreviations are as in Fig. 1

**Fig. 6:**
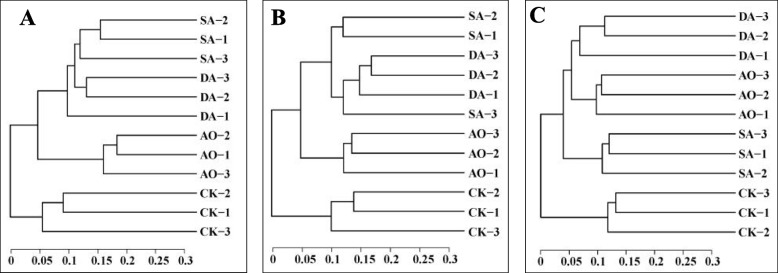
UPGMA clustering analysis based on unweighted unifrac distance matrix at the phylum level. **a** 16S rDNA; (**b**) 18S rDNA; (**c**) ITS. Sample abbreviations are as in Fig. 1

Ordination by principal coordinates analysis (PCoA) of prokaryotes (Fig. 5a) and eukaryotes (Fig. 5b, c) communities was performed to reveal the relationships among the different samples. Based on 16S rDNA sequencing, 18S rDNA sequencing and ITS sequencing, the first principal coordinates axis (PCo1) alone explained 30.37, 35.13 and 30.55% of variance, respectively, and the second principal coordinates axis (PCo2) alone explained 23.72, 20.79 and 15.75% of variance, respectively. The CK samples were grouped on the left-hand side of the graph along PCo1. Figure 6 shows the relationships among the microbial communities in the different samples at the phylum level based on UPGMA method. Both PCoA and UPGMA analysis results showed that the SA and DA samples tended to cluster together based on 16S rDNA sequencing, suggesting that the diseased and decayed algae samples have similar characteristic prokaryotic microorganism communities. And both SA and DA samples were distinct from CK or AO samples based on 16S rDNA sequencing or 18S rDNA sequencing. However, the DA and AO samples tended to cluster together based on ITS sequencing. It implied that the relatedness of eukaryotic community between diseased and decayed algae samples was not in agreement based on different sequencing methods.

### Predicted functional analysis for microbial communities throughTax4Fun and FUNGuild

**Fig. 7:**
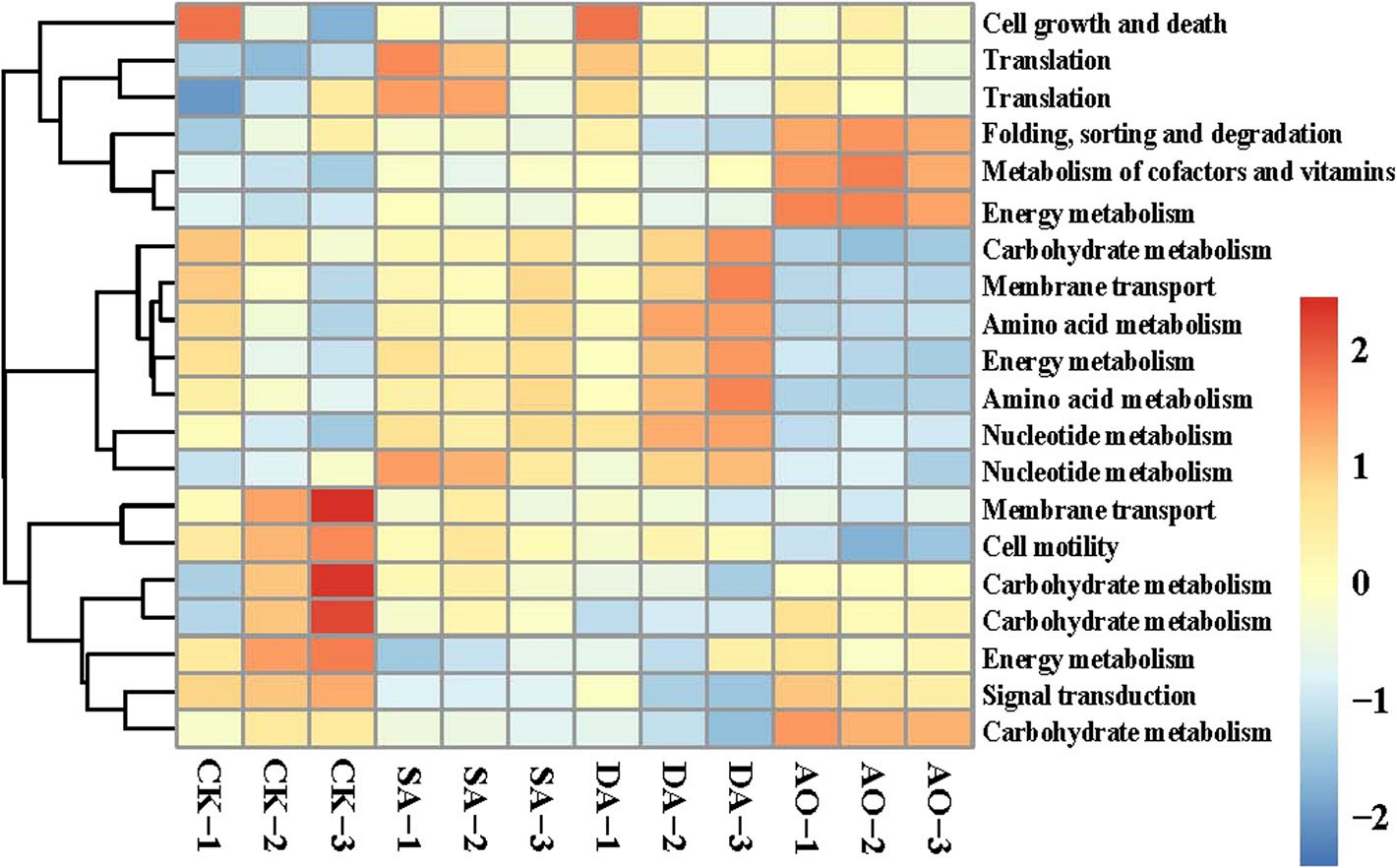
Heat map of the 20 KEGG level-2 functional pathways with relatively high abundance for prokaryotic communities of all samples. The normalized relative abundance of each KEGG pathway is indicated by a gradient of color from blue (low abundance) to red (high abundance). Sample abbreviations are as in Fig. 1

The predicted functional analyses for prokaryotic communities of all samples were carried out using the Tax4Fun tool. Heat map of the 20 KEGG level-2 functional pathways with relatively high abundance are shown in Fig. 7. The predicted functional analysis in all samples found affiliations with metabolic pathways of carbohydrate, energy, nucleotide, amino acids, cofactors and vitamins, and environmental information processing pathways of signal transduction, membrane transport, and genetic information processing pathways of translation, folding, sorting and degradation, and cellular processes pathways of cell motility, cell growth and death. Out of total KEGG subsystems found, carbohydrate metabolism was the highest in abundance in CK, whereas the abundances of nucleotide and amino acids metabolism were found to be significantly lower in CK than in other groups (*p* < 0.05). Moreover, pathways of membrane transport, signal transduction and translation were significantly different in CK compared with those in other groups (p < 0.05).

**Fig. 8:**
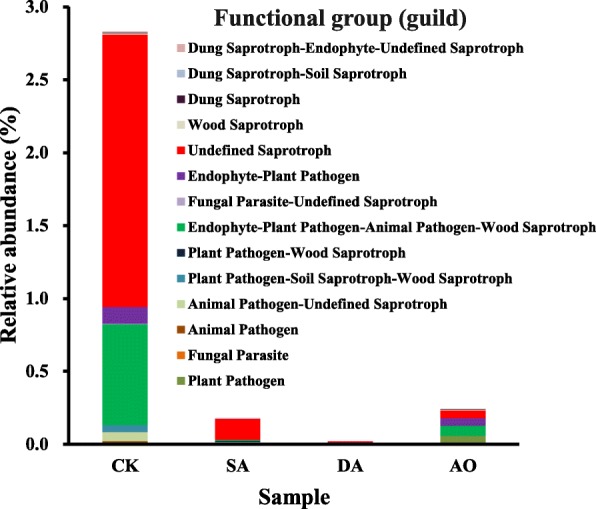
Relative abundance of predicted fungal functions. Unassigned taxa (> 97%) are not shown. Samples abbreviations are as in Fig. 1

The predicted functional analyses for fungal communities in all treatments were carried out using the FUNGuild tool. The relative abundance of 14 fungal functional guilds (not including unassigned taxa) such as plant pathogen, fungal parasite and undefined saprotroph were detected (Fig. 8). The unclassified reads made up a very high proportion (over 97%), reflecting the limitations in the FUNGuild database. The “undefined saprotroph” was the maximum abundance guild followed by “endophyte-plant pathogen-animal pathogen-wood saprotroph” guild, “endophyte-plant pathogen” guild. The above three guilds with relative high abundance accounted for 1.9, 0.7, 0.1% of all sequences respectively in CK, but decreased significantly (*p* < 0.05) in other samples.

## Discussion

### Microbial richness and diversity

Macroalgal surfaces harbor a rich community composed of bacteria, fungi, diatoms, protozoa, spores and larvae of marine invertebrates [40] that can benefit from the availability of various organic substances produced by algae [3]. Bacteria are dominant among primary colonizers [39], whereas fungi appear to be rare in the marine environment [43]. Using high-throughput sequencing, we found that both the richness and diversity of prokaryotic communities were significantly higher than those of eukaryotic communities in all experimental groups in the present study. This implies that prokaryotic organisms are dominant among primary colonizers in the *C. lentillifera* farm. We also found that the microbial richness and diversity among all algae samples were both significantly higher than in the sediment samples, indicating that the microorganisms tended to cluster together around the algae rather than at the bottom of the tank of the *C. lentillifera* farm. Other studies have shown that marine macroalgae are generally associated with specific bacterial communities which differ significantly from those occurring in the surrounding seawater [39, 46]. Both PCoA results and UPGMA results in the present study indicated that among the healthy algal samples, diseased algal samples, and sediment samples, there were different characteristic microbial communities.

Many studies have proposed that there is a mutualistic relationship in which the bacterial community protects the host algae against secondary biological fouling, while the host surface provides nutrients and physical protection to the associated bacteria [57]. Bacteria in a biofilm can affect the growth of other bacteria in the same biofilm [14]. For example, the presence of “resident” bacterial strains on particles either increases or decreases the colonization rate of “newcomer” strains [33]. The accumulative effects of mutualism can facilitate conspecific recruitment and increase the dominance of abundant species, reducing diversity [8]. Generally, 16S rDNA sequencing and 18S rDNA sequencing showed that microbial community diversity was higher in the diseased *C. lentillifera* than in the healthy *C. lentillifera.* Thus, it can be inferred that accumulation of some mutualistic microorganisms may play an important role for the health of *C. lentillifera*. The microbial community diversity was found to be lower in the diseased *C. lentillifera* than in decayed *C. lentillifera* by 16S rDNA sequencing and ITS sequencing, suggesting that a decrease of microbial community diversity may be one of the reasons leading to algal decay. However, the interaction between microbiota and their host is more complex than just a high or low microbial diversity. Thus, no general statements can be made on the role of microbial diversity in health and disease, since different microbe-host interactions are involved in the pathophysiology of different diseases.

### Prokaryotic community composition

Lachnit et al. [40] found that epibacterial community patterns on macroalgae were generally highly host specific but temporally variable. A study by Goecke et al. [30] who isolated bacterial species from more than 42 algal species from marine and freshwater environments found that the two major bacterial groups associated with algae were Bacteroidetes and Proteobacteria, followed by Firmicutes, Actinobacteria, Verrucomicrobia, and Planctomycetes. Yet another study found that bacterial communities belonging to the phyla Proteobacteria and Firmicutes were generally the most abundant on seaweed surfaces [63].

Some bacterial species are considered as an essential functional component of the algal holobiont [61]. Certain physiological properties of bacterial species (i.e. polysaccharide degradation, antibiotic production, growth stimulant production, biosynthesis of allelochemicals, etc.) may favour the establishment of ecological relationships between epibionts and the alga [15, 16].

#### Community composition of prokaryote belonging to the phylum Proteobacteria

Through 16S rDNA sequencing we found that the abundance of Oceanospirillales, *Neptuniibacter* (belonging to Oceanospirillales) and *Aestuariibacter* (belonging to Alteromonadales), belonging to phylum Proteobacteria, were all significantly higher in healthy *C. lentillifera* than in diseased and decayed *C. lentillifera*. Numerous studies have demonstrated the contribution of bacteria to nutrient acquisition or defense by the production of vitamins [74]. Bertrand et al. [9] identified Oceanospirillaceae ASP10-02a as a possible vitamin B_12_ producer in sea-ice edge microbial communities, providing evidence of symbioses between algae and bacteria for vitamin B_12_ acquisition in the natural environment. Thus, it can be inferred that Oceanospirillales and *Neptuniibacter* may contribute to the health of *C. lentillifera* by stimulanting their growth. The most frequently reported bioactive bacterial metabolites have been isolated from species of the genera *Alteromonas*, *Bacillus* and *Pseudoalteromonas*. El Bour et al. [26] isolated *Alteromonas marina* and *Alteromonas macleodii* from *Ulva rigida* and verified that *Alteromonas* showed antibacterial and antifungal bioactivity. *Aestuariibacter* shares many traits with the sister genus *Alteromonas*. Therefore, we proposed that *Aestuariibacter* may favour the health of *C. lentillifera* via antibiotic production.

#### Community composition at the alphaproteobacterial Roseobacter group (Rhodobacteraceae, Proteobacteria)

The abundance of genus *Leisingera*, which belongs to the family Rhodobacteraceae, order Rhodobacterales of the class Alphaproteobacteria, and the abundance of order Rhodospirillales were both significantly lower in healthy *C. lentillifera* than in diseased and decayed *C. lentillifera*. However, the abundance of genus *Labrenzia*, which is the sister genus with *Leisingera*, was higher in healthy *C. lentillifera* than in other samples. The alphaproteobacterial Roseobacter group (Rhodobacteraceae) plays a global role in marine ecosystems with an important role for carbon and sulfur cycling, whose abundance can reach 36% in nutrient-rich costal habitats [52]. It is dominant in the bacterial communities associated with phytoplankton, macroalgae, and various marine animals and both mutualistic and pathogenic life-styles have been suggested [47]. The endophytic bacteria have been microscopically observed in the vacuolar as well as cytoplasmatic regions of various bryopsidalean green algae, including *Bryopsis*, *Halimeda*, and *Caulerpa*. These seaweeds are composed of a single, giant tubular cell and form an interesting biotic environment for bacterial communities [35]. In *Caulerpa* spp., most of the alpha proteobacterial clones were assigned to the Rhodobacteraceae [51]. A number of Rhodobacteraceae organisms are known to produce unique antimicrobial molecules and other secondary metabolites, presenting a potential for detoxication. For example, the genus *Leisingera* can produce the antibacterial compound indigoidine [19, 32]. Moreover, it was revealed that the endosymbiotic Alphaproteobacteria in *Caulerpa* species presented a potential for photosynthesis [22]. For instance, the genus *Labrenzia*, belonging to one kind of aerobic anoxygenic phototrophic bacteria, was able to produce bacteriochlorophyll in small amounts [10]. Therefore, we inferred that there is a symbiotic relationship between *C. lentillifera* and *Leisingera*/*Labrenzia*. On the one hand, *Leisingera* and *Labrenzia* may contribute to the photosynthesis of algae and be favourable for *C. lentillifera* via antibiotic production. On the other hand, significantly increasing *Leisingera* in the bacterial community may not favour the health of the host. Algal diseases usually result from the interaction of environmental factors, pathogen and algae stress response. In certain circumstances, some bacteria produce metabolites and degrade the cell wall of algae. Hence, further investigations for the role of *Leisingera* and *Labrenzia* in the microbial community of *C. lentillifera* should be undertaken.

Biebl et al. (2007) found that *Labrenzia* colonies are white to cream, but may become pink if incubated in the dark under appropriate conditions, and Riedel et al. [60] found that *Leisingera* colonies are dark beige-pink in color. It follows that giving consideration to the color of Rhodobacteraceae colonies and their potential for detoxication, increase in the abundance of Rhodobacteraceae (especially the genus *Leisingera*) may be one of the reasons leading to *C. lentillifera* frond turning pink-red with disease outbreaks. Furthermore, it was frequently observed that the parental frond of *C. lentillifera* would also turn pink-red when they were placed at the bottom of trays under long-term low light conditions. The parental algae’ color changing may be related to Rhodobacteraceae.

#### Community composition of prokaryote belonging to the phylum Planctomycetacia

Using 16S rDNA sequencing, the abundance of Planctomycetales, *Bythopirellula* (Planctomycetes) and *Blastopirellula* (Planctomycetes), were significantly higher in healthy *C. lentillifera* than in diseased and decayed samples of *C. lentillifera*, indicating that Planctomycetes were likely to play a crucial role in the biofilm community of *C. lentillifera*. Through analysis of long chain proteins in the genomes of three Planctomycetes, Faria et al. [28] proposed that Planctomycetes may play an important role in biofilm formation and against stress agents in the complex biofilm of macroalgae. Bengtsson & Øvreås [7] established the importance of Planctomycetes in the biofilm community of the kelp *Laminaria hyperborea*, accounting for 51–53% of the total bacteria. Moreover, several studies also have shown that Planctomycetes appeared frequently in the epibacterial community of macroalgae, presenting clear evidence of an intimate nutritional relationship between Planctomycetes and macroalgae [12, 15, 16, 40, 42, 46]. It has been suggested that the high number of sulfatases found in Planctomycetes could play a major role in the degradation of sulfated polysaccharides in their environment [72]. We therefore speculated that Planctomycetes may also be involved in the utilization of the sulphated polymers produced by the *C. lentillifera*.

#### Community composition of prokaryote belonging to the phyla Bacteroidetes and cyanobacteria

Members of the phylum Bacteroidetes are the most abundant group of bacteria in the ocean after Proteobacteria and Cyanobacteria [29]. It was observed that members of Actinobacteria and Bacteroidetes were the most abundant bacterial species on the surface of *Caulerpa racemosa* [2]. We found that the abundance of Cyanobacteria, Bacteroidetes, and Flavobacteriales (belonging to phylum Bacteroidetes) in diseased and decayed *C. lentillifera* were all significantly higher than in healthy *C. lentillifera*. Moreover, the abundance of Cyanobacteria in the sediment samples was very high, suggesting that the Cyanobacteria outbreak that occurred at the *C. lentillifera* farm may be an important factor causing the disease of *C. lentillifera*. High levels of nitrogen and phosphorus were used in the *C. lentillifera* farm to assist the algae to grow faster. The eutrophic seawater likely provided a suitable environment for the Cyanobacteria outbreak in the farm.

Bacteroidetes also have a close relationship with Cyanobacteria [62, 75]. Bacterial groups such as Cytophagales/Sphingobacteriales (Bacteroidetes), were previously reported to be associated with some harmful algal species [38]. Sphingobacteriales (one of predominant orders in the prokaryotic community of *C. lentillifera*) are known for their ability to degrade toxins and other cyanobacterial secondary metabolites [45]. Moreover, certain members of Sphingobacteriales such as Saprospiraceae, are known to prey on Cyanobacteria [44]. Therefore, some bacterial groups may increase with Cyanobacteria outbreak. There are some bacterial groups which may produce exopolysaccharide substances and extracellular enzymes capable of degrading macromolecules such as cellulose [51]. This likely lead to the spherical ramulis of the infected algae cleaving from the diseased erect branches of *C. lentillifera*.

However, it remains unknown whether the bacteria associated with *C. lentillifera* are beneficial, so their role needs to be clarified. Further investigations will be needed to understand the potential effect of this prokaryotic assemblage on the patterns of *C. lentillifera* colonization.

### Eukaryotic community composition

#### Fungal community composition

Studies based on culturing and molecular methods have shown that Ascomycetes and anamorphic fungi are the predominant endosymbionts of seaweed [34, 78, 79]. The genus *Aspergillus* (belonging to phylum Ascomycota) are adapted to survive as endophytes in marine algae and are prolific producers of novel metabolites having possibly coevolved with the algae [21, 65]. The *Aspergillus* species are common fungal symbionts of many seaweeds including *C. racemosa*, *C. scalpelliformis*, *C. sertularioides*, *Ulva lactuca* and so on [64]. Moreover, *Aspergillusterreus* isolated as an endophyte from *C. scalpelliformis* and *C. sertularioides* can produce insecticidal compounds [65]. We also found that Ascomycota was the dominant fungal phylum detected in *C. lentillifera*. However, the abundance of Ascomycota and *Aspergillus* in diseased *C. lentillifera* was significantly lower than in healthy specimens. Therefore, we hypothesized that Ascomycetes were probably endosymbionts in healthy *C. lentillifera* but the fungal endosymbionts may have difficulty surviving in diseased *C. lentillifera.* Thus, it would be worthwhile determining the role of Ascomycota in stress tolerance and survival of *C. lentillifera*, since endophytes elaborate metabolites or strong antioxidants, making their hosts more resistant to biotic stress such as infection by pathogens [4, 73] or damage by herbivores [71].

#### Epiphytic community composition

The microbial biofilm has been viewed as going through a four-step process: i) adsorption of dissolved organic molecules to a newly submerged surface, ii) colonization of the surface by bacteria, iii) colonization by microscopic eukaryotes (e.g. diatoms, fungi, and other heterotrophic eukaryotes) and iv) settlement and subsequent growth of invertebrate larvae and algal spores [23]. Hence, the establishment of microbial biofilms is regarded as a general prerequisite for the colonization of macroorganisms such as invertebrate larvae and algal spores [13, 56]. However, biofilms also can inhibit larval settlement of marine invertebrates [37]. We found that the abundance of Protozoa and Metazoa on healthy *C. lentillifera* were both significantly higher than those on diseased *C. lentillifera*, suggesting that biofouling with high abundance of Cyanobacteria on diseased *C. lentillifera* was possibly secreting antigrazing compounds into the surrounding seawater to prevent the attachment of grazers.

The external morphology of *C. lentillifera* offers a large three-dimensional substratum on which micro algal propagules my settle. Eutrophic seawater may harbour a number of microalgae and macro algal spores, which can lodge and establish themselves on the surface of *C. lentillifera* and are difficult to dislodge from the host. Eutrophic seawater is likely to provide an ideal environment for an algal propagules outbreak on the farm. We found that the biofouling by Bacillariophyta, Ulvales and *Tetraselmis* on the diseased *C. lentillifera* were much more prolific than those found on the healthy specimens. These biofoulings posed a permanent threat to *C. lentillifera* as they i) increase the hydrodynamic drag on *C. lentillifera*, thereby enhancing the attachment of other fouling organisms, ii) compete for nutrients, iii) inhibit gaseous exchange, and iv) obscure the macroalgae from ambient light.

This study has given insight into how an outbreak of certain epiphytes such as Bacillariophyta, Ulvales and *Tetraselmis*, may be another important factor causing disease in *C. lentillifera*. This conclusion needs more investigation in the future.

### Predicted functional analysis for microbial communities

It was reported that the bacterial community composition on macroalgae is driven by functional genes rather than taxonomic or phylogenetic composition [15, 16]. Moreover, it is also known that the physiological and biochemical properties of the algal host predetermine the composition of the epiphytic bacterial communities. For example, algal cell wall components and secondary metabolites can trigger specific interactions between macroalgae and beneficial bacteria [27, 41]. In the present study, the results of predicted functional analysis for prokaryotic communities implied that algal disease outbreaks significantly changed carbohydrate metabolism, environmental information processing and genetic information processing among prokaryotic communities. FUNGuild analysis showed that there were significantly different fungal functional groupings (or guilds) between healthy and diseased *C. lentillifera* groups. Therefore, we conclude that substantial changes in the physiological and biochemical properties of *C. lentillifera* ensued following the outbreak of algal disease on the farm.

## Conclusions

The prokaryotic and eukaryotic microorganism community structures and diversities in healthy *C. lentillifera* were different from those in diseased *C. lentillifera*. The accumulation of some mutualistic microorganisms may play an important role in the health of *C. lentillifera*. For instance, Oceanospirillales, Neptuniibacter and Aestuariibacter may act as a growth stimulant and in antibiotic production in *C. lentillifera*. There may be a symbiotic relationship between *C. lentillifera* and *Leisingera* and Labrenzia. Ascomycetes were probably endosymbionts in the healthy *C. lentillifera*, whereas the fungal endosymbionts have difficulty surviving in diseased *C. lentillifera*. Epiphytes such as Cyanobacteria, Bacillariophyta, Ulvales and *Tetraselmis* that occurred at the *C. lentillifera* farm sites was an important factor contributing to disease in *C. lentillifera*. These results will provide a theoretical basis for controlling *C. lentillifera* diseases.

## Methods

### Experiment procedure

#### Sample collection

**Fig. 9:**
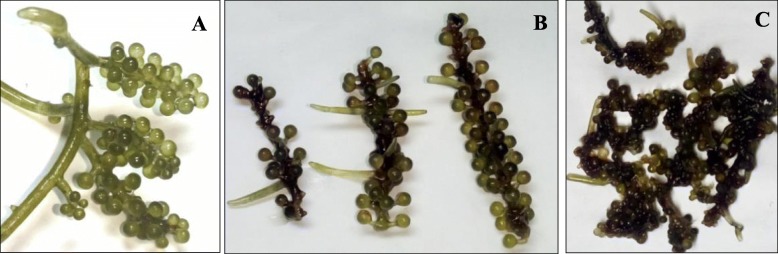
Healthy (**a**), diseased (**b**) and decayed (**c**) samples at the *C. lentillifera* farm in Dalian city in China*.* CK, SA, DA represent the healthy, diseased, and decayed algae samples respectively

Triplicate samples of healthy, diseased, and decayed individuals of *C. lentillifera* were collected from a *C. lentillifera* farm in Changhai county, Dalian city, China, in July 2017. The healthy samples of *C. lentillifera* were analyzed as a control, possessing a bright green color without obvious attachment of other macroscopic fouling organisms (Fig. 9a). The diseased samples were those with obvious biofouling attachments and with some spherical ramulis turning pink-red as well as some missing ramuli from the erect branches (Fig. 9b). The decayed samples are shown in the Fig. 9c.

In addition, triplicate samples of sediment were taken from the bottom of the tank in the *C. lentillifera* farm and were examined for their microbial community structure. Each sample was kept individually enclosed in sterile and sealable plastic bags in situ and transported to the laboratory in a cooler (< 10 °C). Within 4 h after collection, each algal sample was rinsed three times in sterile petri dishes with filtered (0.22 μm pore size) and autoclaved seawater to remove any loosely attached fouling organisms. Subsequently, each sample was gently patted with sterile paper tissue to remove excess seawater and then stored at − 80 °C before nucleic acid analyses. CK, SA, DA respectively represent the healthy, diseased, and decayed algae samples respectively. AO represents the sediment samples collected from the algae farm.

#### DNA extraction and polymerase chain reaction (PCR)

Microbial DNA was extracted from the sample using the E.Z.N.A. DNA Kit (Omega Biotek, Norcross, GA, U.S.) according to the manufacturer’s protocols. The extracted total DNA, dissolved in 30 μl sterile deionized water, was checked by gel-electrophoresis in 1% agarose gel, and its purity was examined by NanoDrop spectrophotometer (NanoDrop Technologies, USA). The variable region V3-V4 of the 16S rDNA and the variable region V4 of the 18S rDNA were selected for the construction of the prokaryotic and eukaryotic community library for Illumina sequencing, respectively. The specific primer set, 341F: 5′-CCTACGGGNGGCWGCAG-3′ and 806R: 5′-GGACTACHVGGGTATCTAAT-3′, was used for amplification of the V3-V4 region of 16S rDNA. And the specific primer set, 515F: 5′-GTGCCAGCMGCCGCGGTAA-3′ and 806R: 5′-GGACTACHVGGGTATCTAAT-3′, was used for amplification of the V4 region of 18S rDNA. The ITS2 region of the ITS rDNA was also selected for the construction of the eukaryotic community library. The specific primer pair ITS3_KYO2F: 5′-GATGAAGAACGYAGYRAA-3′ and ITS4R: 5′-TCCTCCGCTTATTGATATGC-3′ was used for amplification of the ITS2 region. The barcodes in the primers were an eight-base sequence unique to each sample. All amplifications were performed in 50 μl reactions, including 5 μl of template DNA (20 ng/μl), 1.5 μl of each primer (5 μM), 5 μl of each dNTP (2.5 μM), 5 μL of 10 × KOD buffer, and 1 μL of KOD Polymerase. The protocol of amplification was as follows: an initial denaturation at 95 °C for 2 min, followed by 27 cycles of denaturation at 98 °C for 10 s, annealing at 62 °C for 30 s, and elongation at 68 °C for 30 s, and a final extension at 68 °C for 10 min.

#### Illumina Hiseq 2500 sequencing

The amplicons were extracted from 2% agarose gels and purified using the AxyPrep DNA Gel Extraction Kit (Axygen Biosciences, Union City, CA, USA) according to the manufacturer’s instructions and quantified using QuantiFluor-ST (Promega, USA). Purified amplicons were pooled in equimolar and paired-end sequenced (2 × 250) on an Illumina platform according to the standard protocols.

### Statistical and bioinformatics analysis

#### Quality control and reads assembly

Raw data containing adapters or low quality reads would affect the following assembly and analysis. Thus, to get high quality clean reads, quality control and reads assembly were carried out according to the Zhang et al’ study [76] and the following rules: 1) Removing reads containing more than 10% of unknown nucleotides (N); 2) Removing reads containing less than 80% of bases with quality (Q-value) > 20. Paired end clean reads were merged as raw tags using Fast Length Adjustment of Short reads (FLASH) [48] (Version 1.2.11) with a minimum overlap of 10 bp and mismatch error rates of 2%. Noisy sequences of raw tags were filtered by Quantitative Insights Into Microbial Ecology (QIIME) [17] (Version 1.9.1) pipeline under specific filtering conditions [11] to obtain the high-quality clean tags. Clean tags were searched against the reference database (http://drive5.com/uchime/uchime_download.html) to perform reference-based chimera checking using UCHIME algorithm (http://www.drive5.com/usearch/manual/uchime_algo.html). The chimera sequences were finally removed, and the effective tags were generated for further analysis.

#### OTU cluster and taxonomy classification

The effective tags were clustered into operational taxonomic units (OTUs) of ≥ 97% similarity using UPARSE [24] pipeline. The tag sequence with the highest abundance was selected as representative sequence within each cluster. Taxonomic classification of the representative sequence for each OTU was performed using the Ribosomal Database Project classifier (http://rdp.cme.msu.edu/). Each prokaryotic OTU was aligned against SILVA 16S rRNA database (https://www.arb-silva.de/). Each eukaryotic OTU was aligned against the SILVA 18S rRNA database or ITS2 database (http://its2.bioapps.biozentrum.uni-wuerzburg.de/). For prokaryotic OTU analysis, sequences having the best match with eukaryotes (i.e., chloroplasts and mitochondria) were excluded from the OTU table and downstream analyses.

#### Alpha diversity and beta diversity analysis

The coverage percentage was estimated by Good’s method [31]. The abundance-based coverage estimator (ACE), bias-corrected Chao1 richness estimator, and the Shannon and Simpson diversity indices were also calculated in QIIME. OTU rarefaction curve and Rank abundance curves were plotted in QIIME. In the beta diversity analyses, principal coordinate analyses (PCoA) utilizing the unweighted UniFrac distances, were calculated using the R package, and dendrograms were composed using the unweighted pair group method with arithmetic mean (UPGMA) algorithm in BioNumerics to determine the similarity among the samples.

#### Predicted functional analysis for microbial communities

Tax4Fun and FUNGuild analysis were conducted to predict microbial functional profiling. Tax4Fun is an open-source R package that predicts the functional capabilities of prokaryotic communities based on 16SrRNA data sets [5]. And heat map of the Kyoto Encyclopedia of Genes and Genomes (KEGG) level-2 functional pathways was carried out by R package. FUNGuild is a novel tool to comprehensively examine the fungal communities from an ecological perspective [55].

#### Statistical analysis

Data were analyzed using the SPSS 19.0 statistical software packages. All values are presented as the means ± standard deviation (mean ± SD). The level of statistical significance was determined using T-test and Duncan Multiple Comparisons Test. Community composition comparison between two groups was calculated by T-test. Community richness and diversity comparisons among groups were computed by Duncan Multiple Comparisons Test. The statistical significance was set at *p* < 0.05.

## Data Availability

We confirm we have included a statement specifying the local, national or international guidelines and legislation and the required or appropriate permissions and/or licences for the study. Sequence data of this project have been deposited in the Sequence Read Archive (SRA) of the National Center for Biotechnology Information (NCBI) under the accession number PRJNA566062.

## Abbreviations

ITS: Internal transcribed spacer
KEGG: Kyoto Encyclopedia of Genes and Genomes
OTU: Operational Taxonomic Units
PCoA: Principal Coordinate Analyses
UPGMA: Unweighted Pair Group Method With Arithmetic Mean
